# Supplementary material for: Posterior estimation of longitudinal variance components from nonlongitudinal data using Bayesian Gaussian process model
Source: Genetics. 2025 Mar 3;232(1):iyaf036. doi: 10.1093/genetics/iyaf036 (PMC12774850; doi:10.1093/genetics/iyaf036)
Supplement: iyaf036_Supplementary_Data [file iyaf036_supplementary_data.pdf]

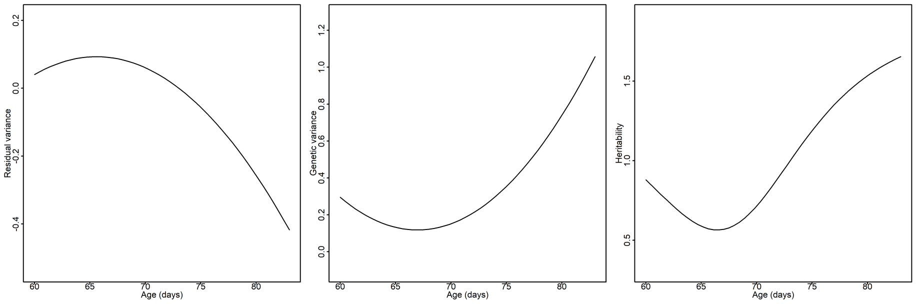

Supplementary Figure 1: Estimated residual and genetic variance components and narrow-sense heritability of HDL cholesterol of mice as a function of age. Random regression model was applied to a dataset of heterogenous stock mice.

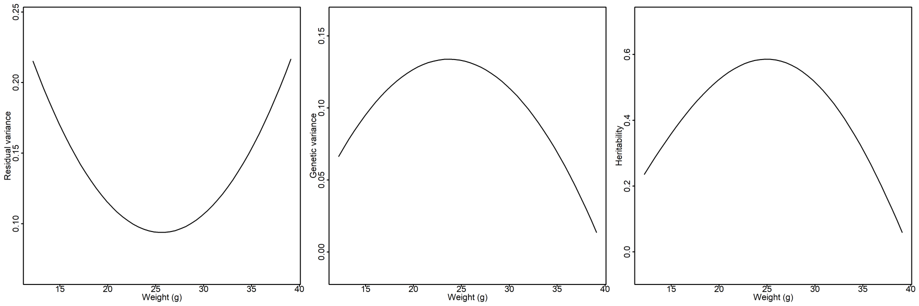

Supplementary Figure 2: Estimated residual and genetic variance components and narrow-sense heritability of HDL cholesterol of mice as a function of weight. Random regression model was applied to a dataset of heterogenous stock mice.

Supplementary Figure 1 plots the variance curves and heritability of stock mice as a function of age, estimated with first-order polynomial random regression model (RRM). The residual variance acquires negative values, which is concerning because we expect variances to be non-negative. This causes the heritability to go over one, which violates the definition of heritability as the proportion of total variance explained by genetic factors. Supplementary Figure 2 shows the variance curves and heritability of stock mice as a function of weight, estimated with first-order polynomial RRM. The heritability curve peaks at 0.6 at about 25 grams of weight.
